# Psychosocial Determinants of Mental Health and Substance Use in Medical Students: A Cross-Sectional Analysis

**DOI:** 10.1192/j.eurpsy.2026.10469

**Published:** 2026-07-31

**Authors:** D. Jarčušková, A. Bednářová, E. Lešťanová

**Affiliations:** 1 1st Department of Psychiatry; 2 2nd Department of Psychiatry; 3 Pavol Jozef Šafárik University, Košice, Slovakia

## Abstract

**Introduction:**

Medical students are exposed to unique psychological stressors that may compromise their mental health. Evidence suggests that they experience higher levels of psychological distress than the general population and their peers in other academic programs.

**Objectives:**

To assess the prevalence of depression, anxiety, stress, substance use, and their associations with attachment style, adverse childhood experiences, and quality of life among medical students.

**Methods:**

We assessed 456 medical students using the Depression, Anxiety and Stress Scale-42 (DASS-42), the Relationship Questionnaire (RQ), the European Health Interview Survey Quality of Life Index (EUROHIS-QOL-8), the CAGE questionnaire, and the Adverse Childhood Experiences International Questionnaire (ACE-IQ).

**Results:**

Depressive symptoms were present in 38.2% of students (image 1), anxiety in 52.6% (image 2), and stress in 42.1% (image 3). Additionally, 22.0% smoked, 4.4% used illicit drugs, and 55.6% reported alcohol use. Stress and anxiety were more common among females, while males more frequently reported alcohol use or alcohol-related problems. Significant associations were found between attachment style and symptoms of depression, anxiety, and stress, as well as between adverse childhood experiences and these outcomes. Poor mental health and substance use were negatively associated with quality of life.

**Image 1:**
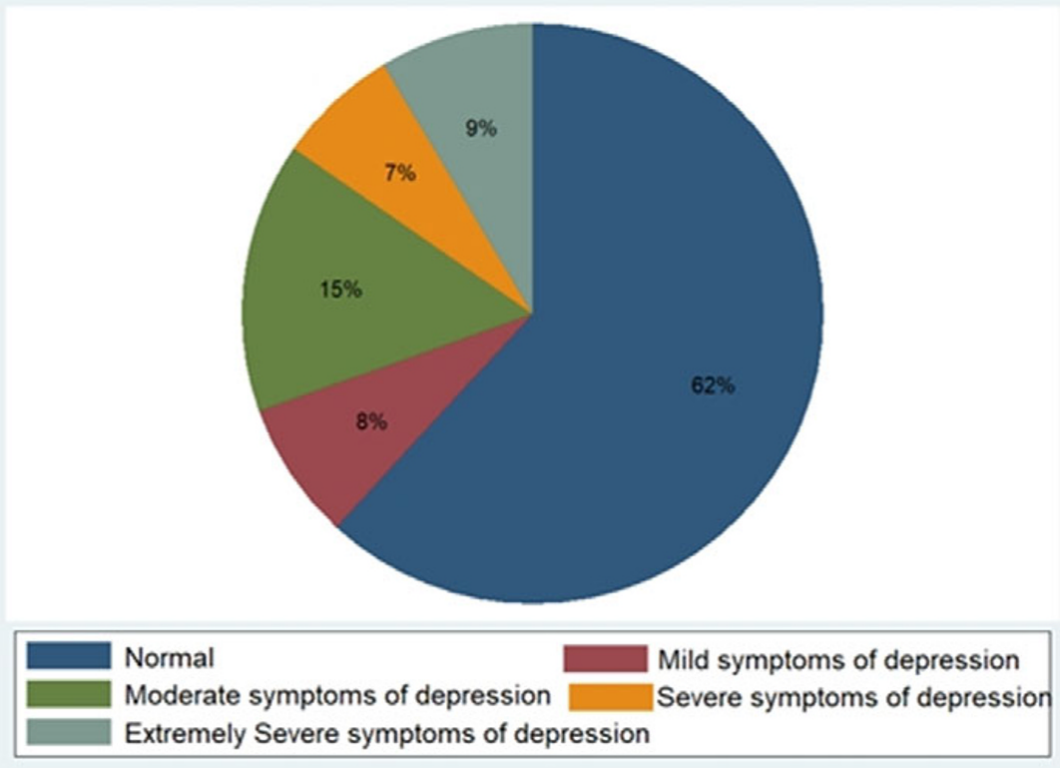

**Image 2:**
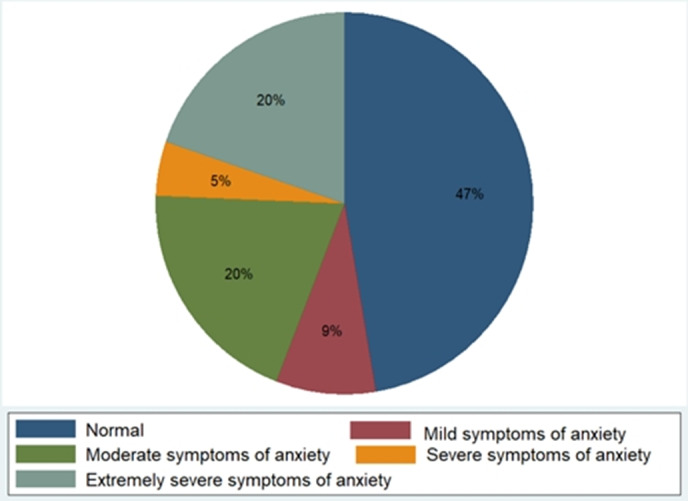

**Image 3:**
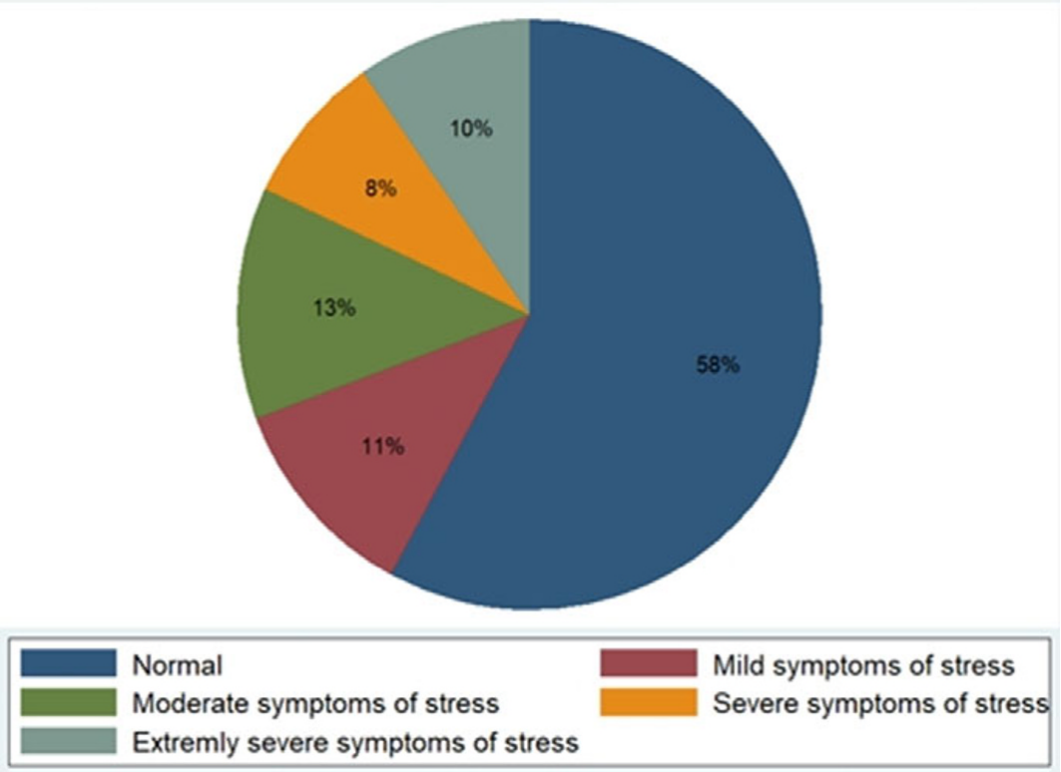

**Conclusions:**

Mental health concerns among medical students are highly prevalent and multifactorial, with serious implications for well-being and professional development. Evidence-based interventions and supportive institutional policies are needed to reduce stigma, promote resilience, and safeguard the future healthcare workforce.

Supported by: Project VEGA 1/0702/25

**Disclosure of Interest:**

None Declared

